# New records of non-native Coleoptera in Italy

**DOI:** 10.3897/BDJ.11.e111487

**Published:** 2023-10-17

**Authors:** Enrico Ruzzier, Lucio Morin, Matteo Zugno, Andrea Tapparo, Luciano Bani, Andrea Di Giulio

**Affiliations:** 1 Department of Science, Roma Tre University, Rome, Italy Department of Science, Roma Tre University Rome Italy; 2 NBFC, National Biodiversity Future Center, Palermo, Italy NBFC, National Biodiversity Future Center Palermo Italy; 3 World Biodiversity Association, Verona, Italy World Biodiversity Association Verona Italy; 4 Regione Lombardia, Laboratorio del Servizio Fitosanitario Regionale c/o Fondazione Minoprio, Vertemate con Minoprio, Italy Regione Lombardia, Laboratorio del Servizio Fitosanitario Regionale c/o Fondazione Minoprio Vertemate con Minoprio Italy; 5 University of Milano-Bicocca, Department of Earth and Environmental Sciences, Milan, Italy University of Milano-Bicocca, Department of Earth and Environmental Sciences Milan Italy

**Keywords:** beetles, biodiversity, Bostrichidae, Cerambycidae, distribution, Monotomidae, Ptinidae, Scolytinae, wood borers

## Abstract

**Background:**

In the last decades, climate change and globalisation have been exacerbating the introduction of non-native beetles worldwide. Due toits peculiar territory, climate and geographical position in the middle of the Mediterranean Basin, Italy is one of the European countries with the highest number of intercepted, adventive and established non-native beetles, some of which are invasive. In this perspective, producing new faunistic records and continuously updating reliable and easily accessible distributional data is a fundamental step in investigating and potentially preventing further species introduction.

**New information:**

The aim of this contribution is to report and discuss new faunistic records of non-native Coleoptera in Italy. For some species, new records enlarge the previously-known distribution (e.g. the ambrosia beetles *Anisandrusmaiche* (Kurentzov, 1941) and *Cnestusmutilatus* (Blandford, 1894) or the click beetle *Monocrepidiusposticus* (Eschscholtz, 1829)), while for others (e.g. the scarab beetle *Archophileurusspinosus* Dechambre, 2006), data confirm their establishment and highlight a possible expansion phase. The false powderpost beetles *Ptilineurusmarmoratus* (Reitter, 1877) and the longhorn beetle *Xylotrechuschinensis* (Chevrolat, 1852) are two new additions to the Italian fauna, while the establishment of the monotomid beetle *Monotomaamericana* Aubé 1837 is confirmed.

## Introduction

In the perspective of an increasingly globalised world in which goods and people move at an ever-increasing speed, maintaining a high level of attention towards non-native and invasive species is fundamental (Banks et al. 2015, Seebens et al. 2018). In particular, together with early detection, the production of new faunistic records and continuous updating of reliable and easily accessible distributional data is a fundamental step in investigating and preventing non-native species introduction and spread (Kenis et al. 2007). Amongst European countries, Italy has one of the highest numbers of intercepted, adventive and established non-native Coleoptera (e.g. Beenen and Roques 2010, Denux and Zagatti 2010, Kirkendall and Faccoli 2010, Roy and Migeon 2010, Sauvard et al. 2010, Yus-Ramos et al. 2014, Nardi et al. 2015, Montagna et al. 2016, Forbicioni 2019, Ruzzier and Colla 2019, Ruzzier et al. 2020b, Ruzzier et al. 2020a, Rattu et al. 2021, Ruzzier et al. 2021c, Ruzzier et al. 2022b, Ruzzier et al. 2023a, Ruzzier et al. 2023c) and this condition is plausibly attributable to Italy's central role in international trade as a crossroads to and from Europe (Roques 2010, Rassati et al. 2014). Given this latter condition, which sees Italy as a possible "gateway to Europe" for non-native species, it is essential to share distribution data to promote an ever-increasing level of attention both in Italy and neighbouring countries against possible natural spreading and introductions. Data collection and monitoring of non-native species, especially if not directed towards specific target taxa of medical, economic or phytosanitary interest, is, however, generally difficult to implement on a national scale; in this regard, the contributions deriving from the non-professional entomological community and citizen-science initiatives prove to be fundamental (e.g. Crall et al. (2010), Larson et al. (2020), Wallace et al. (2021)). This work aims to share faunistic data on Italian non-native beetles in a standard and easily accessible format, updating the distribution or confirming the establishment of some taxa and reporting the arrival of further species.

Beenen and Roques 2010, Denux and Zagatti 2010, Kirkendall and Faccoli 2010, Roy and Migeon 2010, Sauvard et al. 2010, Yus-Ramos et al. 2014, Nardi et al. 2015, Montagna et al. 2016, Ruzzier and Colla 2019, Ruzzier et al. 2020b, Ruzzier et al. 2020a, Rattu et al. 2021, Ruzzier et al. 2021c, Ruzzier et al. 2023a, Ruzzier et al. 2023c

## Materials and methods

Methods for capturing specimens have been quite diverse and included direct collection (e.g. litter/organic debris sifting, beating tray, sweeping net; Iannuzzi et al. (2020)), use of baited traps (Touroult and Witté 2020, Ruchin et al. 2021, Ruzzier et al. 2021a) and light trapping (Hébert et al. 2000, Iannuzzi et al. 2020, Kammar et al. 2020). The collection of data on widespread species was possible thanks to the helpfulness of colleagues and citizens who provided specimens and direct observations (with photos) to the authors. All observations, before being included in the paper, were validated by the first author. Species treated in the "Taxon treatments" section are listed alphabetically.

## Taxon treatments

### Anisandrus maiche

(Kurenzov, 1941)

A0DB8C0F-8548-5334-8666-3705A6F0ECE7

https://www.gbif.org/species/10559613

#### Materials

- **Type status:** Other material. **Occurrence:** individualCount: 1; lifeStage: adult; occurrenceID: EB96945E-1F63-51E5-A39B-8E2194C160F3; **Taxon:** scientificName: *Anisandrusmaiche* (Kurentzov, 1941); family: Curculionidae; **Location:** country: Italy; countryCode: IT; stateProvince: Lombardy; locality: Bosco in città; decimalLatitude: 45.481389; decimalLongitude: 9.099167; geodeticDatum: WGS84; **Identification:** identifiedBy: Enrico Ruzzier; **Event:** eventDate: 2021-05-03
- **Type status:** Other material. **Occurrence:** individualCount: 2; lifeStage: adult; occurrenceID: A17F5897-B862-5285-B77A-E7D52E4F5BAF; **Taxon:** scientificName: *Anisandrusmaiche* (Kurentzov, 1941); family: Curculionidae; **Location:** country: Italy; countryCode: IT; stateProvince: Lombardy; locality: Bosco in città; decimalLatitude: 45.484722; decimalLongitude: 9.088333; geodeticDatum: WGS85; **Identification:** identifiedBy: Enrico Ruzzier; **Event:** eventDate: 2021-05-17
- **Type status:** Other material. **Occurrence:** individualCount: 1; lifeStage: adult; occurrenceID: 95AD5313-4DC9-57D0-9525-D7DBE6D15DC3; **Taxon:** scientificName: *Anisandrusmaiche* (Kurentzov, 1941); family: Curculionidae; **Location:** country: Italy; countryCode: IT; stateProvince: Lombardy; locality: Azzano San Paolo; decimalLatitude: 45.664000; decimalLongitude: 9.678000; geodeticDatum: WGS86; **Identification:** identifiedBy: Enrico Ruzzier; **Event:** eventDate: 2021-05-31
- **Type status:** Other material. **Occurrence:** individualCount: 1; lifeStage: adult; occurrenceID: EFBE387F-891C-556E-B936-495B53D18673; **Taxon:** scientificName: *Anisandrusmaiche* (Kurentzov, 1941); family: Curculionidae; **Location:** country: Italy; countryCode: IT; stateProvince: Lombardy; locality: Bergamo; decimalLatitude: 45.674944; decimalLongitude: 9.683778; geodeticDatum: WGS87; **Identification:** identifiedBy: Enrico Ruzzier; **Event:** eventDate: 2021-05-10
- **Type status:** Other material. **Occurrence:** individualCount: 1; lifeStage: adult; occurrenceID: C7DDD480-4485-507C-8ED4-D6D067D3D956; **Taxon:** scientificName: *Anisandrusmaiche* (Kurentzov, 1941); family: Curculionidae; **Location:** country: Italy; countryCode: IT; stateProvince: Lombardy; locality: Boccaleone; decimalLatitude: 45.684639; decimalLongitude: 9.697556; geodeticDatum: WGS88; **Identification:** identifiedBy: Enrico Ruzzier; **Event:** eventDate: 2021-05-10
- **Type status:** Other material. **Occurrence:** individualCount: 1; lifeStage: adult; occurrenceID: ADB7F3D8-723B-58EB-A63B-0B5760CE90DD; **Taxon:** scientificName: *Anisandrusmaiche* (Kurentzov, 1941); family: Curculionidae; **Location:** country: Italy; countryCode: IT; stateProvince: Lombardy; locality: Boccaleone; decimalLatitude: 45.684639; decimalLongitude: 9.697556; geodeticDatum: WGS88; **Identification:** identifiedBy: Enrico Ruzzier; **Event:** eventDate: 2021-05-25
- **Type status:** Other material. **Occurrence:** individualCount: 4; lifeStage: adult; occurrenceID: 30F1EB94-5082-509D-8C97-3E70DAB5706E; **Taxon:** scientificName: *Anisandrusmaiche* (Kurentzov, 1941); family: Curculionidae; **Location:** country: Italy; countryCode: IT; stateProvince: Lombardy; locality: Boccaleone; decimalLatitude: 45.684639; decimalLongitude: 9.697556; geodeticDatum: WGS88; **Identification:** identifiedBy: Enrico Ruzzier; **Event:** eventDate: 2021-05-31
- **Type status:** Other material. **Occurrence:** individualCount: 1; lifeStage: adult; occurrenceID: 95AE07C1-D5CB-5D42-89CB-0F3B2346F704; **Taxon:** scientificName: *Anisandrusmaiche* (Kurentzov, 1941); family: Curculionidae; **Location:** country: Italy; countryCode: IT; stateProvince: Lombardy; locality: Seriate; decimalLatitude: 45.671583; decimalLongitude: 9.711528; geodeticDatum: WGS88; **Identification:** identifiedBy: Enrico Ruzzier; **Event:** eventDate: 2021-05-31
- **Type status:** Other material. **Occurrence:** individualCount: 3; lifeStage: adult; occurrenceID: 0E4DE7EF-44B6-565A-A939-EF031EAB9939; **Taxon:** scientificName: *Anisandrusmaiche* (Kurentzov, 1941); family: Curculionidae; **Location:** country: Italy; countryCode: IT; stateProvince: Lombardy; locality: Grassobbio; decimalLatitude: 45.662278; decimalLongitude: 9.727806; geodeticDatum: WGS88; **Identification:** identifiedBy: Enrico Ruzzier; **Event:** eventDate: 2021-05-31
- **Type status:** Other material. **Occurrence:** individualCount: 2; lifeStage: adult; occurrenceID: DC4AE596-9ADB-53A0-BB21-03F44A7B18B2; **Taxon:** scientificName: *Anisandrusmaiche* (Kurentzov, 1941); family: Curculionidae; **Location:** country: Italy; countryCode: IT; stateProvince: Lombardy; locality: Seriate; decimalLatitude: 45.667778; decimalLongitude: 9.728417; geodeticDatum: WGS88; **Identification:** identifiedBy: Enrico Ruzzier; **Event:** eventDate: 2021-05-15
- **Type status:** Other material. **Occurrence:** individualCount: 1; lifeStage: adult; occurrenceID: F2EB5C0D-0E3A-51D3-A3B1-FF14C26C2170; **Taxon:** scientificName: *Anisandrusmaiche* (Kurentzov, 1941); family: Curculionidae; **Location:** country: Italy; countryCode: IT; stateProvince: Lombardy; locality: Seriate; decimalLatitude: 45.667778; decimalLongitude: 9.728417; geodeticDatum: WGS88; **Identification:** identifiedBy: Enrico Ruzzier; **Event:** eventDate: 2021-05-31
- **Type status:** Other material. **Occurrence:** individualCount: 3; lifeStage: adult; occurrenceID: 8D2E6670-11B2-5826-A9A7-C2DD7E2B0496; **Taxon:** scientificName: *Anisandrusmaiche* (Kurentzov, 1941); family: Curculionidae; **Location:** country: Italy; countryCode: IT; stateProvince: Lombardy; locality: Seriate; decimalLatitude: 45.671000; decimalLongitude: 9.726000; geodeticDatum: WGS88; **Identification:** identifiedBy: Enrico Ruzzier; **Event:** eventDate: 2021-05-15
- **Type status:** Other material. **Occurrence:** individualCount: 1; lifeStage: adult; occurrenceID: 8BF1294F-CA05-587D-947D-CDED44FDBB13; **Taxon:** scientificName: *Anisandrusmaiche* (Kurentzov, 1941); family: Curculionidae; **Location:** country: Italy; countryCode: IT; stateProvince: Lombardy; locality: Seriate; decimalLatitude: 45.671000; decimalLongitude: 9.726000; geodeticDatum: WGS88; **Identification:** identifiedBy: Enrico Ruzzier; **Event:** eventDate: 2021-05-31
- **Type status:** Other material. **Occurrence:** individualCount: 1; lifeStage: adult; occurrenceID: 9880F35E-E602-584D-9997-6CE2ADB1E853; **Taxon:** scientificName: *Anisandrusmaiche* (Kurentzov, 1941); family: Curculionidae; **Location:** country: Italy; countryCode: IT; stateProvince: Lombardy; locality: Seriate; decimalLatitude: 45.669694; decimalLongitude: 9.729667; geodeticDatum: WGS88; **Identification:** identifiedBy: Enrico Ruzzier; **Event:** eventDate: 2021-05-31
- **Type status:** Other material. **Occurrence:** individualCount: 3; lifeStage: adult; occurrenceID: 97E89BDC-DB16-56A0-A7A0-749B92241D6F; **Taxon:** scientificName: *Anisandrusmaiche* (Kurentzov, 1941); family: Curculionidae; **Location:** country: Italy; countryCode: IT; stateProvince: Lombardy; locality: Seriate; decimalLatitude: 45.652139; decimalLongitude: 9.738250; geodeticDatum: WGS88; **Identification:** identifiedBy: Enrico Ruzzier; **Event:** eventDate: 2021-05-10
- **Type status:** Other material. **Occurrence:** individualCount: 1; lifeStage: adult; occurrenceID: 55BDF733-6A2F-523B-A0EF-191A899A3182; **Taxon:** scientificName: *Anisandrusmaiche* (Kurentzov, 1941); family: Curculionidae; **Location:** country: Italy; countryCode: IT; stateProvince: Lombardy; locality: Seriate; decimalLatitude: 45.652139; decimalLongitude: 9.738250; geodeticDatum: WGS88; **Identification:** identifiedBy: Enrico Ruzzier; **Event:** eventDate: 2021-05-31
- **Type status:** Other material. **Occurrence:** individualCount: 4; lifeStage: adult; occurrenceID: 9B85CEC7-F1D3-59AA-8D3A-3E48B7F4AE60; **Taxon:** scientificName: *Anisandrusmaiche* (Kurentzov, 1941); family: Curculionidae; **Location:** country: Italy; countryCode: IT; stateProvince: Lombardy; locality: Grassobbio; decimalLatitude: 45.653889; decimalLongitude: 9.735000; geodeticDatum: WGS88; **Identification:** identifiedBy: Enrico Ruzzier; **Event:** eventDate: 2021-05-10
- **Type status:** Other material. **Occurrence:** individualCount: 1; lifeStage: adult; occurrenceID: 7A2D9B9B-510F-569B-AC85-270DB67D99CF; **Taxon:** scientificName: *Anisandrusmaiche* (Kurentzov, 1941); family: Curculionidae; **Location:** country: Italy; countryCode: IT; stateProvince: Lombardy; locality: Grassobbio; decimalLatitude: 45.653889; decimalLongitude: 9.735000; geodeticDatum: WGS88; **Identification:** identifiedBy: Enrico Ruzzier; **Event:** eventDate: 2021-05-25
- **Type status:** Other material. **Occurrence:** individualCount: 4; lifeStage: adult; occurrenceID: B094AC4B-3E2F-5B19-A571-5017AF3A3F7A; **Taxon:** scientificName: *Anisandrusmaiche* (Kurentzov, 1941); family: Curculionidae; **Location:** country: Italy; countryCode: IT; stateProvince: Lombardy; locality: Grassobbio; decimalLatitude: 45.653889; decimalLongitude: 9.735000; geodeticDatum: WGS88; **Identification:** identifiedBy: Enrico Ruzzier; **Event:** eventDate: 2021-05-31
- **Type status:** Other material. **Occurrence:** individualCount: 4; lifeStage: adult; occurrenceID: FF811BEC-2DE6-56DA-8281-435A4E7D9A7C; **Taxon:** scientificName: *Anisandrusmaiche* (Kurentzov, 1941); family: Curculionidae; **Location:** country: Italy; countryCode: IT; stateProvince: Lombardy; locality: Grassobbio; decimalLatitude: 45.649500; decimalLongitude: 9.737611; geodeticDatum: WGS88; **Identification:** identifiedBy: Enrico Ruzzier; **Event:** eventDate: 2021-05-25
- **Type status:** Other material. **Occurrence:** individualCount: 1; lifeStage: adult; occurrenceID: 9B3C6161-DDFE-5D39-8B72-5FCFC95AB6AC; **Taxon:** scientificName: *Anisandrusmaiche* (Kurentzov, 1941); family: Curculionidae; **Location:** country: Italy; countryCode: IT; stateProvince: Lombardy; locality: Grassobbio; decimalLatitude: 45.649500; decimalLongitude: 9.737611; geodeticDatum: WGS88; **Identification:** identifiedBy: Enrico Ruzzier; **Event:** eventDate: 2021-05-31
- **Type status:** Other material. **Occurrence:** individualCount: 3; lifeStage: adult; occurrenceID: 2F4E0987-DEE2-553A-AABB-E59A551FD31D; **Taxon:** scientificName: *Anisandrusmaiche* (Kurentzov, 1941); family: Curculionidae; **Location:** country: Italy; countryCode: IT; stateProvince: Lombardy; locality: Peschiera Borromeo; decimalLatitude: 45.438639; decimalLongitude: 9.266667; geodeticDatum: WGS88; **Identification:** identifiedBy: Enrico Ruzzier; **Event:** eventDate: 2021-05-10
- **Type status:** Other material. **Occurrence:** individualCount: 4; lifeStage: adult; occurrenceID: 3A799BDB-3384-5B33-9BB1-2212E6B9518C; **Taxon:** scientificName: *Anisandrusmaiche* (Kurentzov, 1941); family: Curculionidae; **Location:** country: Italy; countryCode: IT; stateProvince: Lombardy; locality: San Donato Milanese; decimalLatitude: 45.423528; decimalLongitude: 9.279444; geodeticDatum: WGS88; **Identification:** identifiedBy: Enrico Ruzzier; **Event:** eventDate: 2021-05-10
- **Type status:** Other material. **Occurrence:** individualCount: 8; lifeStage: adult; occurrenceID: B11939F7-AFCA-5857-B53C-E1E5E8FAC9FA; **Taxon:** scientificName: *Anisandrusmaiche* (Kurentzov, 1941); family: Curculionidae; **Location:** country: Italy; countryCode: IT; stateProvince: Lombardy; locality: Mediglia; decimalLatitude: 45.415778; decimalLongitude: 9.301028; geodeticDatum: WGS88; **Identification:** identifiedBy: Enrico Ruzzier; **Event:** eventDate: 2021-05-10
- **Type status:** Other material. **Occurrence:** individualCount: 2; lifeStage: adult; occurrenceID: 0996BD62-3A20-5F63-84C1-4F692170B483; **Taxon:** scientificName: *Anisandrusmaiche* (Kurentzov, 1941); family: Curculionidae; **Location:** country: Italy; countryCode: IT; stateProvince: Lombardy; locality: Pioltello; decimalLatitude: 45.475611; decimalLongitude: 9.318472; geodeticDatum: WGS88; **Identification:** identifiedBy: Enrico Ruzzier; **Event:** eventDate: 2021-05-10
- **Type status:** Other material. **Occurrence:** individualCount: 1; lifeStage: adult; occurrenceID: 9A7ED195-4A0D-562E-9CFB-4ED98B1BBA27; **Taxon:** scientificName: *Anisandrusmaiche* (Kurentzov, 1941); family: Curculionidae; **Location:** country: Italy; countryCode: IT; stateProvince: Lombardy; locality: San Donato Milanese; decimalLatitude: 45.424583; decimalLongitude: 9.273806; geodeticDatum: WGS88; **Identification:** identifiedBy: Enrico Ruzzier; **Event:** eventDate: 2021-05-10
- **Type status:** Other material. **Occurrence:** individualCount: 1; lifeStage: adult; occurrenceID: 07FEBA84-DD56-5DA6-AB60-445E24AC1298; **Taxon:** scientificName: *Anisandrusmaiche* (Kurentzov, 1941); family: Curculionidae; **Location:** country: Italy; countryCode: IT; stateProvince: Lombardy; locality: La Valletta Brianza; decimalLatitude: 45.714750; decimalLongitude: 9.375583; geodeticDatum: WGS88; **Identification:** identifiedBy: Enrico Ruzzier; **Event:** eventDate: 2021-05-10
- **Type status:** Other material. **Occurrence:** individualCount: 2; lifeStage: adult; occurrenceID: D7FBB323-2EAF-59AD-817B-4B619C619DED; **Taxon:** scientificName: *Anisandrusmaiche* (Kurentzov, 1941); family: Curculionidae; **Location:** country: Italy; countryCode: IT; stateProvince: Lombardy; locality: Montececchia; decimalLatitude: 45.713972; decimalLongitude: 9.353833; geodeticDatum: WGS88; **Identification:** identifiedBy: Enrico Ruzzier; **Event:** eventDate: 2021-05-25
- **Type status:** Other material. **Occurrence:** individualCount: 1; lifeStage: adult; occurrenceID: 10E542AA-430F-5005-B6F5-B12D4A06E9EE; **Taxon:** scientificName: *Anisandrusmaiche* (Kurentzov, 1941); family: Curculionidae; **Location:** country: Italy; countryCode: IT; stateProvince: Lombardy; locality: Montececchia; decimalLatitude: 45.688528; decimalLongitude: 9.373167; geodeticDatum: WGS88; **Identification:** identifiedBy: Enrico Ruzzier; **Event:** eventDate: 2021-05-25
- **Type status:** Other material. **Occurrence:** individualCount: 1; lifeStage: adult; occurrenceID: 483E50C0-637B-588A-9E99-7D58C5E60FE6; **Taxon:** scientificName: *Anisandrusmaiche* (Kurentzov, 1941); family: Curculionidae; **Location:** country: Italy; countryCode: IT; stateProvince: Lombardy; locality: La Valletta Brianza; decimalLatitude: 45.715389; decimalLongitude: 9.375861; geodeticDatum: WGS88; **Identification:** identifiedBy: Enrico Ruzzier; **Event:** eventDate: 2021-05-31

#### Distribution

Eastern Palearctic species, established in European Russia (Terekhova and Skrylnik 2012, Nikulina et al. 2015), Italy (Colombari et al. 2022, Ruzzier et al. 2022b, Mola et al. 2023), North America (Rabaglia et al. 2009, Gomez et al. 2018), Switzerland (Ribeiro-Correia et al. 2023) and Ukraine (Terekhova and Skrylnik 2012, Nikulina et al. 2015).

#### Notes

The data confirm that the species is widely distributed and relatively common in northern Italy, Lombardy in particular. Although *Anisandrusmaiche* is a modestly polyphagous species on broadleaf trees (Ruzzier et al. 2023), no damage attributable to this species has been recorded so far.

### Ambrosiophilus atratus

(Eichhoff, 1875)

C10B17CA-523C-5E97-8552-6D7F3FDFD37E

https://www.gbif.org/species/8046168

#### Materials

- **Type status:** Other material. **Occurrence:** individualCount: 1; occurrenceID: 2085F599-D9B3-55AC-AC25-98DAADC6DE68; **Taxon:** scientificName: *Ambrosiophilusatratus* (Eichhoff 1875); family: Curculionidae; **Location:** country: Italy; countryCode: IT; stateProvince: Lombardia; locality: Pioltello; decimalLatitude: 45.4698309832381; decimalLongitude: 9.32013889739963; geodeticDatum: WGS84; **Identification:** identifiedBy: Enrico Ruzzier; **Event:** eventDate: 2021-04-0

#### Distribution

*Ambrosiophilusatratus* is an ambrosia beetle native to the Eastern Palearctic now established in France, Italy and Slovenia (Faccoli 2008, Dodelin 2019, Hauptman et al. 2019). The present finding is the first record of the species in Lombardy.

#### Notes

*Ambrosiophilusatratus* is a highly polyphagous species, capable of developing in both hardwoods and conifers (Ruzzier et al. 2023). Recent observations, coupled with the lack of damage attributable to the species, however, seem to suggest that *A.atratus* might have a negligible phytosanitary value, preferring severely debilitated or already dead plants as reproductive substrate (Ranger et al. 2010, Reed et al. 2015, Ruzzier et al. 2022a).

### Archophileurus spinosus

Dechambre, 2006

2567B555-8259-5BEC-B6B6-9E4C4F9647EC

https://www.gbif.org/species/1073568

#### Materials

- **Type status:** Other material. **Occurrence:** individualCount: 1; sex: male; occurrenceID: 38D8EE03-F4E1-5701-9B44-666FBCA56537; **Taxon:** scientificName: *Archophileurusspinosus* Dechambre, 2006; family: Scarabaeidae; **Location:** country: Italy; countryCode: IT; county: Puglia; locality: Molfetta; decimalLatitude: 41.184601; decimalLongitude: 16.622004; geodeticDatum: WGS8; **Identification:** identifiedBy: Enrico Ruzzier; **Event:** eventDate: 2020-07-09
- **Type status:** Other material. **Occurrence:** individualCount: 1; sex: female; occurrenceID: B785307E-E50B-51B3-9466-21E2066A1E5C; **Taxon:** scientificName: *Archophileurusspinosus* Dechambre, 2006; family: Scarabaeidae; **Location:** country: Italy; countryCode: IT; county: Puglia; locality: Minervino di Lecce; decimalLatitude: 40.090350; decimalLongitude: 18.431938; geodeticDatum: WGS8; **Identification:** identifiedBy: Enrico Ruzzier; **Event:** eventDate: 2023-07-20

#### Notes

The finding of the specimen in Molfetta, more than 170 km from the area of the first recording (see Ruzzier et al. (2020b)), would suggest the existence of a second population of this species, probably as a result of an independent introduction. The specimen collected in Minervino di Lecce may also be the result of an independent introduction or be confirmation that the species is expanding in southern Apulia. The fact that the specimens were observed in nature suggests the existence of at least two naturalised populations.

### Caplothorax lugubris

(Murray, 1864)

0B0344EA-32CA-53F3-BB0E-28F7AF064005

https://www.gbif.org/species/1044108

#### Materials

- **Type status:** Other material. **Occurrence:** individualCount: 1; occurrenceID: C12A2CD3-F6F7-5357-B897-91B6AEC1B840; **Taxon:** scientificName: *Caplothoraxlugubris* (Murray, 1864); family: Nitidulidae; **Location:** country: Italy; countryCode: IT; stateProvince: Veneto; locality: Cassanego (Borso del Grappa); decimalLatitude: 45.827633; decimalLongitude: 11.811687; geodeticDatum: WGS84; **Identification:** identifiedBy: Enrico Ruzzier; **Event:** eventDate: 2021-03-04
- **Type status:** Other material. **Occurrence:** individualCount: 1; occurrenceID: 34E90A5F-BF19-5172-948A-D29A0F7D461D; **Taxon:** scientificName: *Caplothoraxlugubris* (Murray, 1864); family: Nitidulidae; **Location:** country: Italy; countryCode: IT; stateProvince: Veneto; locality: Pederobba; decimalLatitude: 45.884568; decimalLongitude: 11.944527; geodeticDatum: WGS84; **Event:** eventTime: 2021-04-17
- **Type status:** Other material. **Occurrence:** individualCount: 4; occurrenceID: 7710809D-DDF0-548A-A92C-E920A3CE75AA; **Taxon:** scientificName: *Caplothoraxlugubris* (Murray, 1864); family: Nitidulidae; **Location:** country: Italy; countryCode: IT; stateProvince: Friuli Venezia Giulia; locality: Ronchi dei Legionari; decimalLatitude: 45.824606; decimalLongitude: 13.495001; geodeticDatum: WGS8; **Identification:** identifiedBy: Enrico Ruzzier; **Event:** eventDate: 2019-08-20
- **Type status:** Other material. **Occurrence:** individualCount: 1; occurrenceID: 7D090E7D-F9DB-5881-B1C9-AF8B35A35A99; **Taxon:** scientificName: *Caplothoraxlugubris* (Murray, 1864); family: Nitidulidae; **Location:** country: Italy; countryCode: IT; stateProvince: Friuli Venezia Giulia; locality: Monte Calvario; decimalLatitude: 45.952522; decimalLongitude: 13.583972; geodeticDatum: WGS84; **Identification:** identifiedBy: Enrico Ruzzier; **Event:** eventDate: 2019-04-03
- **Type status:** Other material. **Occurrence:** individualCount: 7; occurrenceID: 4F4A32F2-0773-5411-B774-BA7361D59E98; **Taxon:** scientificName: *Caplothoraxlugubris* (Murray, 1864); family: Nitidulidae; **Location:** country: Italy; countryCode: IT; stateProvince: Friuli Venezia Giulia; locality: Monte Calvario; decimalLatitude: 45.952522; decimalLongitude: 13.583972; geodeticDatum: WGS8; **Identification:** identifiedBy: Enrico Ruzzier; **Event:** eventDate: 2019-05-09
- **Type status:** Other material. **Occurrence:** individualCount: 1; occurrenceID: 51B94B3C-5C87-5CF1-B4DF-7515EB58E368; **Taxon:** scientificName: *Caplothoraxlugubris* (Murray, 1864); family: Nitidulidae; **Location:** country: Italy; countryCode: IT; stateProvince: Veneto; locality: Creazzo; decimalLatitude: 45.527977; decimalLongitude: 11.488399; geodeticDatum: WGS84; **Identification:** identifiedBy: Enrico Ruzzier; **Event:** eventDate: 2019-11-09

#### Distribution

Species of Nearctic and Neotropical distribution (Parsons 1943), established in Italy since 2013 (Marini et al. 2013, Jelínek et al. 2016).

#### Notes

Species of phytosanitary relevance (e.g. Harrison (1962), Dowd (2000), Marini et al. (2013), Jelínek et al. (2016)) now widely distributed in the Po plain (Northern Italy), were is frequently injurious on *Zeamays* L. and *Fragaria* sp. (authors' personal observations).

### Cnestus mutilatus

(Blandford, 1894)

AAD440AB-E85C-5FD7-B59F-B8F346F331BC

#### Materials

- **Type status:** Other material. **Occurrence:** recordedBy: Enrico Ruzzier; individualCount: 1; sex: female; occurrenceID: 867EB1BE-5F06-5C36-BFA3-378BF566184B; **Taxon:** scientificName: *Cnestusmutilatus* (Blandford, 1894); family: Curculionida; **Location:** country: Italy; countryCode: IT; stateProvince: Veneto; municipality: Borso del Grappa; decimalLatitude: 45.815456; decimalLongitude: 11.769814; geodeticDatum: WGS84; **Identification:** identifiedBy: Enrico Ruzzier; **Event:** eventDate: 2023-08-08

#### Distribution

Species native to the Oriental and Eastern Palearctic Regions, established in North America (Gomez et al. 2018, Smith et al. 2020) and Italy (Colombari et al. 2022, Marchioro et al. 2022). This finding further extends the distribution range of the species.

#### Notes

*Cnestusmutilatus* is a somewhat polyphagous species (Ruzzier et al. 2023b), including plants of economic/productive relevance (e.g. Mandelshtam et al. (2019), Ruzzier et al. (2021b), Monterrosa et al. (2022)), yet its host plants in Europe were not known. The specimen on which the report is based was found in a tunnel bored into a branch of an unidentified conifer (probably *Abies*). This is the first observation in which *C.mutilatus* was found in association with a conifer; however, it is plausible that this plant might not be a reproductive host.

### Dinoderus (Dinoderastes) japonicus

Lesné, 1895

D7E3D2EF-87CA-5637-8F19-D5B83DAB45A4

https://www.gbif.org/species/4427616

#### Materials

- **Type status:** Other material. **Occurrence:** recordedBy: Lucio Morin; individualCount: 1; occurrenceID: 3FDA78E1-BEA0-5E32-AB07-6E9C35C04C2F; **Taxon:** scientificName: *Dinoderusjaponicus* Lesné, 1895; family: Bostrichidae; **Location:** country: Italy; countryCode: IT; stateProvince: Friuli Venezia Giulia; locality: Ronchi dei Legionari; decimalLatitude: 45.824285; decimalLongitude: 13.495115; geodeticDatum: WGS84; **Identification:** identifiedBy: Enrico Ruzzier; **Event:** eventDate: 2020-05-1
- **Type status:** Other material. **Occurrence:** recordedBy: Enrico Ruzzier; individualCount: 1; occurrenceID: BDCD3FCA-A8F4-508F-A50E-FBEE98D010A5; **Taxon:** scientificName: *Dinoderusjaponicus* Lesné, 1895; family: Bostrichidae; **Location:** country: Italy; countryCode: IT; stateProvince: Veneto; locality: Altavilla Vicentina; decimalLatitude: 45.521738; decimalLongitude: 11.478075; geodeticDatum: WGS84; **Identification:** identifiedBy: Enrico Ruzzier; **Event:** eventDate: 2021-08-05

#### Distribution

Species native to China, Japan, Korea and Taiwan, recorded multiple times in Austria, France, Germany, Great Britain, Italy, Poland, Sweden, Switzerland and the Netherlands; observations indicate established populations only in France (Brustel and Aberlenc 2014), Italy (Nardi et al. 2015) and, more recently, in the Caucasus (Bieńkowski and Orlova-Bienkowskaja 2017).

#### Notes

The recollection of the single specimen in Ronchi dei Legionari, in nature, suggests the existence of an established population in the area. However, given the close vicinity of the Monfalcone port, a well-known port of entry for non-native species (Rassati et al. 2014), it might be possible that the specimen would be the result of a recent introduction. The specimen recollected in Altavilla Vicentina was found already dead inside a DIY store, suggesting its introduction via bamboo products.

### Monocrepidius posticus

(Eschscholtz, 1829)

759161A0-631B-5659-8FDF-274DE430CD51

https://www.gbif.org/species/6984492

#### Materials

- **Type status:** Other material. **Occurrence:** recordedBy: Rudolf Schuh; individualCount: 26; occurrenceID: D85425C8-4501-55CA-8092-190FD66AB239; **Taxon:** scientificName: *Monocrepidiusposticus* (Eschscholtz, 1829); family: Elateridae; **Location:** country: Italy; countryCode: IT; stateProvince: Calabria; locality: Torre Mezza Praia, Acconia; decimalLatitude: 38.8162444606245; decimalLongitude: 16.2220915973993; geodeticDatum: WGS84; **Identification:** identifiedBy: Rudolf Schuh; **Event:** eventDate: 2014-06-28
- **Type status:** Other material. **Occurrence:** recordedBy: Giovanni Altadonna; individualCount: 1; occurrenceID: 3178C064-E00D-5267-8230-DAE8EEA1D0C8; **Taxon:** scientificName: *Monocrepidiusposticus* (Eschscholtz, 1829); family: Elateridae; **Location:** country: Italy; countryCode: IT; stateProvince: Sicily; county: Messina; locality: Tremestieri; decimalLatitude: 38.143639; decimalLongitude: 15.522750; geodeticDatum: WGS84; **Identification:** identifiedBy: Giovanni Altadonna; **Event:** eventDate: 2023-06-28
- **Type status:** Other material. **Occurrence:** recordedBy: Giovanni Altadonna; individualCount: 1; occurrenceID: 5D51C8AA-856B-5E00-87EA-BBB20B575E39; **Taxon:** scientificName: *Monocrepidiusposticus* (Eschscholtz, 1829); family: Elateridae; **Location:** country: Italy; countryCode: IT; stateProvince: Sicily; county: Messina; locality: Tremestieri; decimalLatitude: 38.143639; decimalLongitude: 15.522750; geodeticDatum: WGS84; **Identification:** identifiedBy: Giovanni Altadonna; **Event:** eventDate: 2023-07-08
- **Type status:** Other material. **Occurrence:** recordedBy: Enrico Ruzzier; individualCount: 1; occurrenceID: E628CAD2-F417-5A7D-B164-644C000CC0E5; **Taxon:** scientificName: *Monocrepidiusposticus* (Eschscholtz, 1829); family: Elateridae; **Location:** country: Italy; countryCode: IT; stateProvince: Veneto; county: Vicenza; locality: Costalunga; decimalLatitude: 45.811018; decimalLongitude: 11.743925; geodeticDatum: WGS84; **Identification:** identifiedBy: Enrico Ruzzier; **Event:** eventDate: 2023-08-12

#### Distribution

Species native to the Neotropics and now established in both the Nearctic and Western Palearctic (Ruzzier et al. 2021c, Van Meer and Artéro 2022). *Monocrepidiusposticus* is seemingly a species of relatively recent introduction that, however, has been able to spread in peninsular Italy and Sicily. The rapid diffusion of this species is most probably associated with potted plants (Ruzzier et al. 2021c), especially when the insect is at the larval stage. Specimens from Calabria and Veneto represent new regional records that further demonstrate the wide distribution of the species on the Italian territory.

#### Notes

Species included in the CABI Invasive Species Compendium; to date, there is no information on the real economic or environmental impact of this non-native species (Ruzzier et al. 2021c). This species was earlier known as *Conoderusposticus*; however, the name *Conoderus* Eschscholtz, 1829 is a synonym of *Monocrepidius* Eschscholtz, 1829 (see Kundrata et al. (2019)).

### Monotoma americana

Aubé, 1837

252CC0B0-F04D-5B5E-9E8C-BB80A6E44C73

#### Materials

- **Type status:** Other material. **Occurrence:** recordedBy: Lucio Morin; individualCount: 100; lifeStage: adult; occurrenceID: 555B1798-5F9E-5831-B495-B95E460B5E6D; **Taxon:** scientificName: Monotomaamericana Aubé 1837; family: Monotomida; **Location:** country: Italy; stateProvince: Friuli Venezia Giulia; county: Gorizia; municipality: Rochi dei Legionari; decimalLatitude: 45.838596; decimalLongitude: 13.497632; geodeticDatum: WGS84; **Identification:** identifiedBy: Enrico Ruzzier; **Event:** eventDate: 2020-11-20

#### Distribution

North American species (Bousquet and Laplante 1999) introduced in Croatia and Italy (Jelínek 2007).

#### Notes

The finding of additional specimens confirms the establishment of this species in the Italian territory, as the occurrence of this taxon was so far limited to the only vague indication given in Jelínek (2007).

### Ptilineurus marmoratus

(Reitter, 1877)

A535107E-DDE6-5593-A431-F2AC6CF0E91E

https://www.gbif.org/species/1095559

#### Materials

- **Type status:** Other material. **Occurrence:** recordedBy: Davide Pedersoli; individualCount: 1; occurrenceID: 9D84AA13-EAE2-55A3-9AEB-1DDC4358CABE; **Taxon:** scientificName: *Ptilineurusmarmoratus* (Reitter, 1877); family: Ptinidae; **Location:** country: Italy; countryCode: IT; stateProvince: Lombardy; county: Brescia; municipality: Esine; decimalLatitude: 45.92741; decimalLongitude: 10.25646; geodeticDatum: WGS84; **Identification:** identifiedBy: Enrico Ruzzier; **Event:** eventDate: 2022-06-12
- **Type status:** Other material. **Occurrence:** recordedBy: Luca Cristiano; individualCount: 1; occurrenceID: 1CFB379D-F5FA-5665-915E-07E7C5FD86E3; **Taxon:** scientificName: *Ptilineurusmarmoratus* (Reitter, 1877); family: Ptinidae; **Location:** country: Italy; countryCode: IT; stateProvince: Piedmont; county: Asti; municipality: Rocchetta Tanaro; decimalLatitude: 44.8622778; decimalLongitude: 8.3246666; geodeticDatum: WGS84; **Identification:** identifiedBy: Enrico Ruzzier; **Event:** eventDate: 2022-06-30

#### Distribution

Taxon native to China, Indonesia, Japan and introduced in Germany, Sweden and USA (Zahradník 2012, Zahradník 2021, Jarzabek-Müller 2023); new species for the Italian fauna.

#### Notes

The two records, which occurred in different regions and in nature, clearly indicate the establishment of the species in the Italian territory. *Ptilineurusmarmoratus* is a negligible pest in the countries of origin, where it has been recorded damaging stored grains (Yan et al. 2010) or furnishings (Yuasa and Onoe 1930).

### Psacothea hilaris

(Pascoe, 1857)

A904EACB-F8F9-54AD-A48E-28CCC0703294

https://www.gbif.org/species/9166077

#### Materials

- **Type status:** Other material. **Occurrence:** individualCount: 1; occurrenceID: CBDF2EE5-F84F-5374-9DB7-B5E0247C1B51; **Taxon:** scientificName: *Psacotheahilaris* (Pascoe, 1857); family: Cerambycidae; **Location:** country: Italy; countryCode: IT; stateProvince: Lombardia; locality: Carugate; decimalLatitude: 45.551858579102; decimalLongitude: 9.3466620693504; geodeticDatum: WGS8; **Identification:** identifiedBy: Enrico Ruzzier; **Event:** eventDate: 2021-08-25
- **Type status:** Other material. **Occurrence:** individualCount: 1; occurrenceID: E6FE9FF5-BE9F-54F0-82EE-55C401085FC7; **Taxon:** scientificName: *Psacotheahilaris* (Pascoe, 1857); family: Cerambycidae; **Location:** country: Italy; countryCode: IT; stateProvince: Lombardia; locality: Carate Brianza; decimalLatitude: 45.6792254670083; decimalLongitude: 9.22969294016464; geodeticDatum: WGS84; **Identification:** identifiedBy: Enrico Ruzzier; **Event:** eventDate: 2021-08-13
- **Type status:** Other material. **Occurrence:** individualCount: 1; occurrenceID: 7ABA8012-C57A-57BD-9709-726A4DEEB99C; **Taxon:** scientificName: *Psacotheahilaris* (Pascoe, 1857); family: Cerambycidae; **Location:** country: Italy; countryCode: IT; stateProvince: Lombardia; locality: Mandello del Lario; decimalLatitude: 45.9141449791127; decimalLongitude: 9.32590399764125; geodeticDatum: WGS84; **Identification:** identifiedBy: Enrico Ruzzier; **Event:** eventDate: 2021-08-15
- **Type status:** Other material. **Occurrence:** individualCount: 1; occurrenceID: 63207C90-E03D-565D-AB6B-011CF79005D6; **Taxon:** scientificName: *Psacotheahilaris* (Pascoe, 1857); family: Cerambycidae; **Location:** country: Italy; countryCode: IT; stateProvince: Lombardia; locality: Valbrona; decimalLatitude: 45.8757857966675; decimalLongitude: 9.2992147264757; geodeticDatum: WGS84; **Identification:** identifiedBy: Enrico Ruzzier; **Event:** eventDate: 2021-08-13
- **Type status:** Other material. **Occurrence:** individualCount: 1; occurrenceID: 113CAE03-455D-560A-B0C4-5D471A19FD07; **Taxon:** scientificName: *Psacotheahilaris* (Pascoe, 1857); family: Cerambycidae; **Location:** country: Italy; countryCode: IT; stateProvince: Lombardia; locality: Viganò; decimalLatitude: 45.715744797865; decimalLongitude: 9.32365682691914; geodeticDatum: WGS84; **Identification:** identifiedBy: Enrico Ruzzier; **Event:** eventDate: 2021-07-31
- **Type status:** Other material. **Occurrence:** individualCount: 1; occurrenceID: 876A5500-62E0-5609-BE88-85B64AD9BA81; **Taxon:** scientificName: *Psacotheahilaris* (Pascoe, 1857); family: Cerambycidae; **Location:** country: Italy; countryCode: IT; stateProvince: Lombardia; locality: Valaperta; decimalLatitude: 45.6731438758896; decimalLongitude: 9.35553589763243; geodeticDatum: WGS84; **Identification:** identifiedBy: Enrico Ruzzier; **Event:** eventDate: 2020-07-20
- **Type status:** Other material. **Occurrence:** individualCount: 1; occurrenceID: AFCB9F02-0C7C-5DFC-A0BE-1BE208E1BC31; **Taxon:** scientificName: *Psacotheahilaris* (Pascoe, 1857); family: Cerambycidae; **Location:** country: Italy; countryCode: IT; stateProvince: Lombardia; locality: Casetello di Brianza; decimalLatitude: 45.764033; decimalLongitude: 9.34946156924904; geodeticDatum: WGS84; **Identification:** identifiedBy: Enrico Ruzzier; **Event:** eventDate: 2021-06-09
- **Type status:** Other material. **Occurrence:** individualCount: 1; occurrenceID: 236111F8-A763-515A-993E-1FA25C760478; **Taxon:** scientificName: *Psacotheahilaris* (Pascoe, 1857); family: Cerambycidae; **Location:** country: Italy; countryCode: IT; stateProvince: Lombardia; locality: Nesso; decimalLatitude: 45.91001879; decimalLongitude: 9.15568364012023; geodeticDatum: WGS84; **Identification:** identifiedBy: Enrico Ruzzier; **Event:** eventDate: 2019-09-02
- **Type status:** Other material. **Occurrence:** individualCount: 1; occurrenceID: 6D4BCC00-4E11-5109-8352-FB4803E70DB9; **Taxon:** scientificName: *Psacotheahilaris* (Pascoe, 1857); family: Cerambycidae; **Location:** country: Italy; countryCode: IT; stateProvince: Lombardia; locality: Castelmarte; decimalLatitude: 45.8327755037426; decimalLongitude: 9.24120299763827; geodeticDatum: WGS84; **Event:** eventDate: 2019-08-3
- **Type status:** Other material. **Occurrence:** individualCount: 1; occurrenceID: 3AC5E866-5647-5D50-A8CD-AD9D46110B4D; **Taxon:** scientificName: *Psacotheahilaris* (Pascoe, 1857); family: Cerambycidae; **Location:** country: Italy; countryCode: IT; stateProvince: Lombardia; locality: Sala di Calolziocorte; decimalLatitude: 45.7906744601323; decimalLongitude: 9.44158372647262; geodeticDatum: WGS84; **Identification:** identifiedBy: Enrico Ruzzier; **Event:** eventDate: 2020-06-25
- **Type status:** Other material. **Occurrence:** individualCount: 1; occurrenceID: 8B6CBB74-10B6-59BE-B51B-3B07CC764CD6; **Taxon:** scientificName: *Psacotheahilaris* (Pascoe, 1857); family: Cerambycidae; **Location:** country: Italy; countryCode: IT; stateProvince: Lombardia; locality: Como; decimalLatitude: 45.7826544507253; decimalLongitude: 9.08968049763644; geodeticDatum: WGS84; **Identification:** identifiedBy: Enrico Ruzzier; **Event:** eventDate: 2021-08-30
- **Type status:** Other material. **Occurrence:** individualCount: 1; occurrenceID: 5DEBB24C-1B93-5D45-8A44-7BBA731F4D11; **Taxon:** scientificName: *Psacotheahilaris* (Pascoe, 1857); family: Cerambycidae; **Location:** country: Italy; countryCode: IT; stateProvince: Lombardia; locality: Calolziocorte; decimalLatitude: 45.7916760173374; decimalLongitude: 9.44862099763678; geodeticDatum: WGS84; **Identification:** identifiedBy: Enrico Ruzzier; **Event:** eventDate: 2020-10-25
- **Type status:** Other material. **Occurrence:** individualCount: 1; occurrenceID: 3FBFE588-631C-5034-A930-AC6A11E9B40E; **Taxon:** scientificName: *Psacotheahilaris* (Pascoe, 1857); family: Cerambycidae; **Location:** country: Italy; countryCode: IT; stateProvince: Lombardia; locality: Villasanta; decimalLatitude: 45.617095; decimalLongitude: 9.299486; geodeticDatum: WGS84; **Identification:** identifiedBy: Enrico Ruzzier; **Event:** eventDate: 2023-07-17
- **Type status:** Other material. **Occurrence:** individualCount: 1; occurrenceID: 7E650CF7-A0E1-51A4-BCC9-1E6CBE8862A7; **Taxon:** scientificName: *Psacotheahilaris* (Pascoe, 1857); family: Cerambycidae; **Location:** country: Italy; countryCode: IT; stateProvince: Lombardia; locality: Cernusco dul Naviglio; decimalLatitude: 45.522631; decimalLongitude: 9.338841; geodeticDatum: WGS84; **Identification:** identifiedBy: Enrico Ruzzier; **Event:** eventDate: 2023-07-14
- **Type status:** Other material. **Occurrence:** individualCount: 1; occurrenceID: E8910DA7-1194-5217-8D26-65E321CD7FED; **Taxon:** scientificName: *Psacotheahilaris* (Pascoe, 1857); family: Cerambycidae; **Location:** country: Italy; countryCode: IT; stateProvince: Lombardia; locality: Castelnuovo; decimalLatitude: 45.692475; decimalLongitude: 9.322235; geodeticDatum: WGS84; **Identification:** identifiedBy: Enrico Ruzzier; **Event:** eventDate: 2023-06-09
- **Type status:** Other material. **Occurrence:** individualCount: 1; occurrenceID: AD01E2C1-1FF7-5AC9-9C1C-6C7E14760A34; **Taxon:** scientificName: *Psacotheahilaris* (Pascoe, 1857); family: Cerambycidae; **Location:** country: Italy; countryCode: IT; stateProvince: Lombardia; locality: Arlate; decimalLatitude: 45.729312; decimalLongitude: 9.441062; geodeticDatum: WGS84; **Identification:** identifiedBy: Enrico Ruzzier; **Event:** eventDate: 2023-07-29

#### Distribution

Species widely distributed in southeast China and Japan (Danilevsky 2020), introduced and established in Italy (Lupi et al. 2013).

#### Notes

*Psacotheahilaris* is facing a substantial expansion phase in northern Italy (Lupi et al. 2023), where it is a pest on *Ficuscarica* L. (Moracee) (Lupi et al. 2013).

### Sinoxylon unidentatum

(Fabricius, 1801)

DC83C1FE-9949-5D92-9A6E-9D07DB583EC8

https://www.gbif.org/species/6005764

#### Materials

- **Type status:** Other material. **Occurrence:** individualCount: 1; occurrenceID: 676575BD-B096-510A-A62F-8C24806832C6; **Taxon:** scientificName: *Sinoxylonunidentatum* (Fabricius, 1801); family: Bostrichida; **Location:** country: Italy; countryCode: IT; stateProvince: Veneto; locality: Marghera - Porto; decimalLatitude: 45.4675788; decimalLongitude: 12.237859; geodeticDatum: WGS84; **Identification:** identifiedBy: Enrico Ruzzier; **Event:** eventDate: 2021-09-27

#### Distribution

Species of Oriental origin and now established in Asia, North, Central and South America, Africa (including Madagascar) and Oceania (Lykidis et al. 2016).

#### Notes

*Sinoxylonunidentatum* has been recorded multiple times in Europe (Nardi and Mifsud 2015, Lykidis et al. 2016), although it appears that there are currently no established populations (Lykidis et al. 2016). The fact that the specimen presented here was collected in close proximity to a port area would suggest its recent arrival rather than the existence of a stable population.

### Stelidota geminata

(Say, 1825)

7BD3522E-C7E3-51B1-9B97-27392B54592A

https://www.gbif.org/species/1043978

#### Materials

- **Type status:** Other material. **Occurrence:** individualCount: 5; occurrenceID: 5551F673-ECC8-5945-9549-80EE22205D8D; **Taxon:** scientificName: *Stelidotageminata* (Say, 1825); family: Nitidulidae; **Location:** country: Italy; countryCode: IT; stateProvince: Veneto; locality: Cismon del Grappa; decimalLatitude: 45.930615; decimalLongitude: 11.731266; **Identification:** identifiedBy: Enrico Ruzzier; **Event:** eventTime: 2021-02-15
- **Type status:** Other material. **Occurrence:** individualCount: 1; occurrenceID: 5BEB32C2-4714-515E-865D-9434470A652D; **Taxon:** scientificNameID: *Stelidotageminata* (Say, 1825); family: Nitidulidae; **Location:** country: Italy; countryCode: IT; stateProvince: Veneto; locality: Madonnetta (Pieve del Grappa); decimalLatitude: 45.850090; decimalLongitude: 11.838718; geodeticDatum: WGS84; **Identification:** identifiedBy: Enrico Ruzzier; **Event:** eventDate: 2021-03-04
- **Type status:** Other material. **Occurrence:** individualCount: 1; occurrenceID: F865FB4A-7A60-56CC-B7A4-7BCAF2B56908; **Taxon:** scientificName: *Stelidotageminata* (Say, 1825); family: Nitidulidae; **Location:** country: Italy; countryCode: IT; stateProvince: Veneto; locality: Cavaso del Tomba; decimalLatitude: 45.87854064067142; decimalLongitude: 11.870694397663117; geodeticDatum: WGS84; **Identification:** identifiedBy: Enrico Ruzzier; **Event:** eventTime: 2020-10-31
- **Type status:** Other material. **Occurrence:** individualCount: 1; occurrenceID: BA8E0D16-9738-5D36-8E02-CD458527A1B4; **Taxon:** scientificName: *Stelidotageminata* (Say, 1825); family: Nitidulidae; **Location:** country: Italy; countryCode: IT; stateProvince: Veneto; locality: Romano d'Ezzelino; decimalLatitude: 45.79608863822578; decimalLongitude: 11.744732926495166; geodeticDatum: WGS84; **Identification:** identifiedBy: Enrico Ruzzier; **Event:** eventDate: 2020-10-01
- **Type status:** Other material. **Occurrence:** individualCount: 1; occurrenceID: 95FE36BB-B51F-5361-AB40-1EB21FEE1E16; **Taxon:** scientificName: *Stelidotageminata* (Say, 1825); family: Nitidulidae; **Location:** country: Italy; countryCode: IT; stateProvince: Veneto; locality: Val dei Ponti (Solagna); decimalLatitude: 45.824872; decimalLongitude: 11.719725; geodeticDatum: WGS84; **Identification:** identifiedBy: Enrico Ruzzier; **Event:** eventDate: 2021-04-29
- **Type status:** Other material. **Occurrence:** individualCount: 1; occurrenceID: FD7E0C67-C3CD-59FD-AC56-92718EDDD617; **Taxon:** scientificName: *Stelidotageminata* (Say, 1825); family: Nitidulidae; **Location:** country: Italy; countryCode: IT; stateProvince: Calabria; locality: Torre Mezza Praia, Acconia; decimalLatitude: 38.8162444606245; decimalLongitude: 16.2220915973993; geodeticDatum: WGS84; **Identification:** identifiedBy: Rudolf Schuh; **Event:** eventDate: 2014-062
- **Type status:** Other material. **Occurrence:** individualCount: 23; occurrenceID: 345F5E0E-D792-53A0-B3CC-38D9CE0972F2; **Taxon:** scientificName: *Stelidotageminata* (Say, 1825); family: Nitidulidae; **Location:** country: Italy; countryCode: IT; stateProvince: Veneto; locality: San Piero di Feletto; decimalLatitude: 45.9296259207839; decimalLongitude: 12.2343788273258; geodeticDatum: WGS84; **Identification:** identifiedBy: Enrico Ruzzier; **Event:** eventDate: 2021-08-13
- **Type status:** Other material. **Occurrence:** individualCount: 1; occurrenceID: 3A918067-EA6F-5E90-94EE-35B7AD7B52D7; **Taxon:** scientificName: *Stelidotageminata* (Say, 1825); family: Nitidulidae; **Location:** country: Italy; countryCode: IT; stateProvince: Friuli Venezia Giulia; locality: Marina Julia (Monfalcone); decimalLatitude: 45.780855; decimalLongitude: 13.522679; geodeticDatum: WGS84; **Identification:** identifiedBy: Lucio Morin; **Event:** eventDate: 2017-03-17
- **Type status:** Other material. **Occurrence:** individualCount: 1; occurrenceID: DF66CDE3-D4A6-5F85-9588-45DCEC391444; **Taxon:** scientificName: *Stelidotageminata* (Say, 1825); family: Nitidulidae; **Location:** country: Italy; countryCode: IT; stateProvince: Friuli Venezia Giulia; locality: Piedimonte- Monte Calvario; decimalLatitude: 45.944862; decimalLongitude: 13.583999; geodeticDatum: WGS84; **Identification:** identifiedBy: Lucio Morin; **Event:** eventDate: 2020-05-23
- **Type status:** Other material. **Occurrence:** occurrenceID: 92C5CB23-6FB3-541F-9643-7B7BD1A48741; **Taxon:** scientificName: *Stelidotageminata* (Say, 1825); family: Nitidulidae; **Location:** country: Italy; countryCode: IT; stateProvince: Friuli Venezia Giulia; locality: Duino - Bosco della Cernizza; decimalLatitude: 45.777; decimalLongitude: 13.594; geodeticDatum: WGS84; **Identification:** identifiedBy: Lucio Morin; **Event:** eventDate: 2011-11-27
- **Type status:** Other material. **Occurrence:** individualCount: 2; occurrenceID: B48C6458-0EB2-549A-B42E-13AF551B7D0D; **Taxon:** scientificName: *Stelidotageminata* (Say, 1825); family: Nitidulidae; **Location:** country: Italy; countryCode: IT; stateProvince: Veneto; locality: Creazzo; decimalLatitude: 45.5460; decimalLongitude: 11.4780; geodeticDatum: WGS84; **Identification:** identifiedBy: Enrico Ruzzier; **Event:** eventDate: 2015-04-25
- **Type status:** Other material. **Occurrence:** individualCount: 2; occurrenceID: EE28EC36-B058-57D3-BBE0-C9AA2E589258; **Taxon:** scientificName: *Stelidotageminata* (Say, 1825); family: Nitidulidae; **Location:** country: ITALY; countryCode: IT; stateProvince: Lombardia; locality: Angone; decimalLatitude: 45.905485; decimalLongitude: 10.198955; geodeticDatum: WGS84; **Identification:** identifiedBy: Davide Pedersoli; **Event:** eventDate: 2006-09-06
- **Type status:** Other material. **Occurrence:** occurrenceID: CA1227D5-3BFC-5A44-8DD7-DFF162590571; **Taxon:** scientificName: *Stelidotageminata* (Say, 1825); family: Nitidulidae; **Location:** country: Italy; countryCode: IT; stateProvince: Lombardia; locality: Boario; decimalLatitude: 45.891122; decimalLongitude: 10.189661; geodeticDatum: WGS84; **Identification:** identifiedBy: Davide Pedersoli; **Event:** eventDate: 2008-08-24
- **Type status:** Other material. **Occurrence:** individualCount: 1; occurrenceID: D8A9309C-5293-5450-940F-B50FA1CFE040; **Taxon:** scientificName: *Stelidotageminata* (Say, 1825); family: Nitidulidae; **Location:** country: Italy; countryCode: IT; stateProvince: Lombardia; locality: Bunote di Angone; decimalLatitude: 45.911237; decimalLongitude: 10.204144; geodeticDatum: WGS84; **Identification:** identifiedBy: Davide Pedersoli; **Event:** eventDate: 2012-05-08
- **Type status:** Other material. **Occurrence:** individualCount: 1; occurrenceID: 27691415-EFCE-5316-8EC4-3DAD5BBA921F; **Taxon:** scientificName: *Stelidotageminata* (Say, 1825); family: Nitidulidae; **Location:** country: Italy; countryCode: IT; stateProvince: Lombardia; locality: Esine; decimalLatitude: 45.905999; decimalLongitude: 10.222361; geodeticDatum: WGS84; **Identification:** identifiedBy: Davide Pedersoli; **Event:** eventDate: 2016-06-25
- **Type status:** Other material. **Occurrence:** individualCount: 1; occurrenceID: 2A18DEB8-174F-53B8-8BC5-1251AC0BACEF; **Taxon:** scientificName: *Stelidotageminata* (Say, 1825); family: Nitidulidae; **Location:** country: Italy; countryCode: IT; stateProvince: Lombardia; locality: Tisa; decimalLatitude: 46.180218; decimalLongitude: 10.318497; geodeticDatum: WGS84; **Identification:** identifiedBy: Davide Pedersoli; **Event:** eventTime: 2017-08-0

#### Distribution

Species of North American origin and established in Central and South America, Europe, the Oriental Region and in the Near East (Peck et al. 2014, Jelínek et al. 2016, Stan 2019).

#### Notes

*Stelidotageminata* is an relevant pest of strawberries in North America (Weiss and Williams 1980, Loughner et al. 2007). This species is expanding rapidly all over the world and, in Europe, it was reported at first in the Azores in the 1980s (Israelson 1985) and then in several continental countries (Jelínek et al. 2016, Guéorguiev 2018, Stan 2019, Wojas 2022). In Italy, it seems acclimatised since the second half of the 1990s (Ratti 2002), where it has been recorded as phytosaprophagous (Pezzi 2013), but also as a concurrent source of damage on cultivated strawberries (Pansa et al. 2014, author's personal observations).

### Telephanus atricapillus

Erichson, 1846

A0B04073-6900-5D5B-8273-B25D98915A9F

https://www.gbif.org/species/1043900

#### Materials

- **Type status:** Other material. **Occurrence:** recordedBy: Nicola Roncen; individualCount: 1; occurrenceID: E12C0E6A-B19C-5386-A74F-E5AF2317312C; **Taxon:** scientificName: *Telephanusatricapillus* Erichson, 1846; family: Silvanidae; **Location:** country: Italy; countryCode: IT; stateProvince: Veneto; locality: Vellai (Feltre); decimalLatitude: 46.035593; decimalLongitude: 11.942838; geodeticDatum: WGS8; **Identification:** identifiedBy: Nicola Roncen; **Event:** eventDate: 2012-07-07
- **Type status:** Other material. **Occurrence:** recordedBy: Nicola Roncen; individualCount: 1; occurrenceID: 37AB006F-63EE-5E7A-8D13-4667D1CA79B7; **Taxon:** scientificName: *Telephanusatricapillus* Erichson, 1846; family: Silvanidae; **Location:** country: Italy; countryCode: IT; stateProvince: Veneto; locality: Vellai (Feltre); decimalLatitude: 46.032920; decimalLongitude: 11.951203; geodeticDatum: WGS84; **Identification:** identifiedBy: Nicola Roncen; **Event:** eventDate: 2012-07-14
- **Type status:** Other material. **Occurrence:** recordedBy: Lucio Morin; individualCount: 1; occurrenceID: 8577D6E8-230A-5976-98A3-EB5DAEC4F4D3; **Taxon:** scientificName: *Telephanusatricapillus* Erichson, 1846; family: Silvanidae; **Location:** country: Italy; countryCode: IT; stateProvince: Friuli Venezia Giulia; locality: San Pier d'Isonzo; decimalLatitude: 45.851512; decimalLongitude: 13.453215; geodeticDatum: WGS84; **Identification:** identifiedBy: Lucio Morin; **Event:** eventDate: 2020-05-16
- **Type status:** Other material. **Occurrence:** individualCount: 1; occurrenceID: 8D59979F-4A95-5FD4-8DE0-2C18D71F3A88; **Taxon:** scientificName: *Telephanusatricapillus* Erichson, 1846; family: Silvanidae; **Location:** country: Italy; countryCode: IT; stateProvince: Friuli Venezia Giulia; locality: San Pier d'Isonzo; decimalLatitude: 45.851512; decimalLongitude: 13.453215; geodeticDatum: WGS84; **Identification:** identifiedBy: Lucio Morin; **Event:** eventDate: 2020-05-18
- **Type status:** Other material. **Occurrence:** individualCount: 1; occurrenceID: 71DEFE24-F2B6-5609-BD00-C767F8395DED; **Taxon:** scientificName: *Telephanusatricapillus* Erichson, 1846; family: Silvanidae; **Location:** country: Italy; countryCode: IT; stateProvince: Friuli Venezia Giulia; locality: San Canzian D'Isonzo; decimalLatitude: 45.769977; decimalLongitude: 13.471999; geodeticDatum: WGS84; **Identification:** identifiedBy: Lucio Morin; **Event:** eventDate: 2021-03-01
- **Type status:** Other material. **Occurrence:** recordedBy: Enrico Ruzzier; individualCount: 1; occurrenceID: 3F1B1E10-333D-591A-B4A8-6214A5563E09; **Taxon:** scientificName: *Telephanusatricapillus* Erichson, 1846; family: Silvanidae; **Location:** country: Italy; countryCode: IT; stateProvince: Veneto; locality: Romano d'Ezzelino; decimalLatitude: 45.813867; decimalLongitude: 11.754639; geodeticDatum: WGS8; **Identification:** identifiedBy: Enrico Ruzzier; **Event:** eventDate: 2023-05-06

#### Distribution

Species of Nearctic origin, established both in Italy and Spain (Ruzzier et al. 2020a). The records here presented substantially extend the distribution of *T.atricapillus* in northern Italy.

### Trichoferus campestris

(Faldermann, 1835)

329B7A17-44C6-5D20-9CF0-4EBE7D8AEBB8

https://www.gbif.org/species/1151427

#### Materials

- **Type status:** Other material. **Occurrence:** individualCount: 1; occurrenceID: F3CD819B-D612-5D95-BAE2-0F92550A96FD; **Taxon:** scientificName: *Trichoferuscampestris* (Faldermann, 1835); family: Cerambycidae; **Location:** country: Italy; countryCode: IT; stateProvince: Veneto; locality: Saonara; decimalLatitude: 45.394167; decimalLongitude: 12.007500; geodeticDatum: WGS8; **Identification:** identifiedBy: Enrico Ruzzier; **Event:** eventDate: 2018-07-03

#### Distribution

Species of Eastern Palearctic origin, introduced in Europe and North America (Danilevsky 2020, Cocquempot et al. 2022).

#### Notes

In Italy, *Trichoferuscampestris* was first detected in Piacenza in 2000 (Cocquempot et al. 2022) and subsequently intercepted in Naples in 2015 (Pennacchio et al. 2016), recorded in Veneto (2018, this paper) and recently observed in Lombardy in 2020 (Cocquempot et al. 2022, source INaturalist). It would seem that all the specimens observed belong to single introductions and that, currently, no established populations exist on the Italian territory. However, this situation requires further investigation given the phytosanitary relevance of the species (Allen and Humble 2002, EFSA Panel on Plant Health et al. 2021).

### Xylotrechus chinensis

(Chevrolat, 1852)

DB77E5F4-0C0E-51DF-B027-AB66E6A9DBB4

https://www.gbif.org/species/8325707

#### Materials

- **Type status:** Other material. **Occurrence:** individualCount: 1; occurrenceID: 898AAD92-D272-5BC8-8BBB-06C6C605BECA; **Taxon:** scientificName: *Xylotrechuschinensis* (Chevrolat, 1852); family: Cerambycidae; **Location:** country: Italy; countryCode: IT; stateProvince: Lombardy; municipality: Roè Volciano; locality: Tormini; decimalLatitude: 45.607926; decimalLongitude: 10.4682366; geodeticDatum: WGS84; **Identification:** identifiedBy: Enrico Ruzzier; **Event:** eventDate: 2023-07-02
- **Type status:** Other material. **Occurrence:** individualCount: 1; occurrenceID: 932B446C-0D4C-545A-9CE6-6AAB60E5ED00; **Taxon:** scientificName: *Xylotrechuschinensis* (Chevrolat, 1852); family: Cerambycidae; **Location:** country: Italy; countryCode: IT; stateProvince: Lombardy; municipality: Prevalle; decimalLatitude: 45.553290; decimalLongitude: 10.422359; geodeticDatum: WGS84; **Identification:** identifiedBy: Enrico Ruzzier; **Event:** eventDate: 2023-06-10
- **Type status:** Other material. **Occurrence:** individualCount: 1; occurrenceID: BE3DA5D1-FF92-5169-B3A6-637BD4D2F519; **Taxon:** scientificName: *Xylotrechuschinensis* (Chevrolat, 1852); family: Cerambycidae; **Location:** country: Italy; countryCode: IT; stateProvince: Lombardy; municipality: Muscoline; decimalLatitude: 45.572222; decimalLongitude: 10.482320; geodeticDatum: WGS84; **Identification:** identifiedBy: Enrico Ruzzier; **Event:** eventDate: 2023-06-23

#### Distribution

Species native to East Asia, introduced in Europe (Leivadara et al. 2018, Sarto i Monteys and Torras i Tutusaus 2018, Cocquempot et al. 2019); new species for the Italian fauna.

#### Notes

In Europe, *Xylotrechuschinensis* was first officially reported in 2013 in Catalonia (Sarto i Monteys and Torras i Tutusaus 2018) and subsequently was observed to be established also in Greece (Leivadara et al. 2018) and France (Cocquempot et al. 2019). The three records here presented and observed in the Province of Brescia (Lombardy) indicate the first record of *X.chinensis* in Italy. The species was promptly notified to the Regional Plant Health Service (Regione Lombardia Plant Health Service) and to the European Commission (Europhyt Outbreak No. 2250). Several records of exit holes and adults on plants of *Morus* sp. clearly indicate the establishment of *X.chinensis* on the Italian territory. There are currently no signs of attack on the other host plants known from literature (Han and Lyu 2010) and present in the Italian invaded area, such as *Malus* spp., *Pyrus* spp. and *Vitisvinifera* L. (Europhyt Outbreak No. 2250).

## Discussion

The faunistic records here presented further demonstrated how Italy, due to its climatic and environmental peculiarities, is rather predisposed to host and foster the establishment of non-native beetles; in confirmation, it is sufficient to note that most of the species that have arrived in recent decades are all undergoing a substantial range expansion. It is also interesting to note that species new to the Italian fauna are already present at the European level, indicating a general homogenisation of the non-native fauna due to species natural spreading and unintentional human-mediated transport amongst EU countries. The latter could be the case for the recent arrival of *Xylotrechuschinensis*, whose Italian population may originate from infested areas in Spain or France, rather than a random introduction from countries of origin (a hypothesis that requires further investigation). Given the enormous diversity of species and adaptations that characterise Coleoptera, maintaining a national level of monitoring and data collection would not be possible without the continued support of the non-professional entomological community, as well as from citizen-science initiatives. In particular, especially for species that are more conspicuous and identifiable by photography, at least in the first instance, careful monitoring of social networks can help to collect totally unexpected observations.

## Supplementary Material

XML Treatment for Anisandrus maiche

XML Treatment for Ambrosiophilus atratus

XML Treatment for Archophileurus spinosus

XML Treatment for Caplothorax lugubris

XML Treatment for Cnestus mutilatus

XML Treatment for Dinoderus (Dinoderastes) japonicus

XML Treatment for Monocrepidius posticus

XML Treatment for Monotoma americana

XML Treatment for Ptilineurus marmoratus

XML Treatment for Psacothea hilaris

XML Treatment for Sinoxylon unidentatum

XML Treatment for Stelidota geminata

XML Treatment for Telephanus atricapillus

XML Treatment for Trichoferus campestris

XML Treatment for Xylotrechus chinensis
